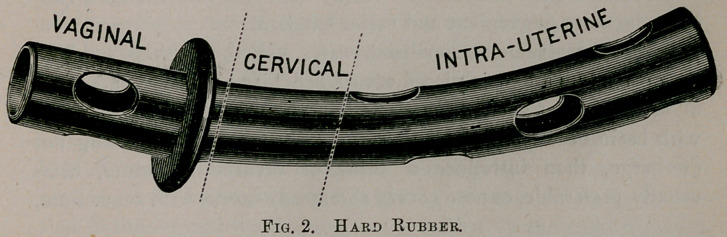# Indications for and Aseptic Technique of Uterine Drainage after Labor and Abortion*Read at the meeting of the Georgia Medical Association, April 20, 1898.

**Published:** 1898-07

**Authors:** R. R. Kime

**Affiliations:** Atlanta, Ga.


### INDICATIONS FOR AND ANTISEPTIC TECHNIQUE OF UTERINE DRAINAGE AFTER LABOR AND ABORTION.*

By R. R. KIME, M.D.,

Atlanta, Ga.

When we consider that at least thirty to fifty per cent. of diseases of women are traceable, directly or indirectly, to injuries or accidents during or after labor and abortions, that fully one-half of that number are due to infection in some one of its various forms, we need no apology for presenting this subject.

While some would consider "puerperal fever," so-called, a threadbare subject, and others would fain relegate it to oblivion by saying it is a preventable disease—that the physician is responsible for all such cases—nevertheless it is always with us, and many a woman suffers from its ravages.

Volumes have been written, many experiments made, many lives lost, and the question is far from being solved to-day.

Semelweiss and Holmes began investigations and promulgated views which have been reinforced by the germ theory of disease and rendered fertile by the antiseptic and aseptic era, and now stand out as landmarks in the prevention and relief of puerperal infection.

Life-saving measures have thus been evolved which may ultimately lead to more accurate scientific methods in the prevention and relief of puerperal infection and its consequent results. As an evidence of the unsettled condition, we have but to briefly review the methods of treatment advised and practiced for the prevention and relief of these conditions.

Some use ante-partum douches, some use post-partum douches, others condemn both. Some use antiseptic methods of prevention, some aseptic methods, others none whatever. Some advise washing out the uterus with strong antiseptics in puerperal infection, some use weak antiseptic solutions, others condemn the use of any. Some advise curetting uterus in all varieties of infection, some curette

*Read at the meeting of the Georgia Medical Association, April 20, 1898.

only in putrid infection (sapremia), others condemn the curette in all cases. Some advise abdominal section in severe cases, some perform hysterectomy, others condemn such operative measures and resort to vaginal drainage by incision as a life-saving measure. Some advise stimulants in large quantities, some depend upon strychnia, quinine and nourishment, while others kill their patients with the coal-tar group as antipyretics. Some use the serum treatment, some the nucleins, others fail to get good results from either. Some administer opiates in full doses, some advise salines, while the author favors elimination and drainage.

Happy is the man that can judiciously select and apply to each individual case such treatment as will not only save life, but conserve the generative organs for future physiological functions.

In the treatment of puerperal infection we must consider the anatomical relations and physiological functions of the female generative organs, the direct communication between uterine and pelvic cavities by way of the fallopian tubes, the intimate relations of the pelvic organs by the lymphatic and vascular channels, rendering the female peculiarly susceptible to the invasion of infection.

When we consider the great changes wrought in the generative organs during pregnancy, and the nearer to full term the greater the size of the uterus, more vascular blood supply, greater activity of lymphatics, with consequent retrograde changes necessary to complete involution after emptying uterus, we have indications which should direct our treatment of these cases.

An infected uterus after full-term labor is large, flabby, relaxed, will not drain itself however patulous the os, does not contract so as to empty cavity and shut off the avenues of infection.

The placental sight, condition of endometrium, and retrograde tissue changes necessary to involution, require, even demand, a process of elimination or drainage from uterine cavity, even in normal cases; much more so when infection occurs.

When we have putrid infection, septic infection or pus formation in other parts of the body, the rule is to open up, disinfect, wash out and drain. Why should the uterus be an exception to this rule?

Nature establishes a flow, current of elimination, the lochia after labor; obstruct that channel of elimination, and what is the result? This discharge unloads the endometrium, relieves the lymphatics, throws off toxins, débris and germs that would otherwise be retained or absorbed and cause trouble.

I do not believe all cases of puerperal infection are due to contamination by physician or nurse, nor that the physician can always prevent such infection.

We certainly have sufficient clinical evidence to prove that where a uterus fails to properly drain itself (which often occurs), remaining large and flabby, a blood-clot, portions of placenta or a cotyledon is retained, infection occurs from putrefaction and absorption of uterine contents. Such cases are usually sapremia or putrid infection, but may be a mixed or true septic infection, due to presence of septic or pus-producing germs in genital tract previous to confinement.

It is in the cases of sapremia (putrid infection) where some physicians get good results from the use of curette, antiseptic douche and gauze tampon, because the offending material is removed, absorption checked and uterus stimulated to contraction.

In septic infection the question is more serious, as the curette and gauze *tampon* have killed more patients than they have saved.

It is time the authors of our text-books on obstetrics were recognizing the dangers of such treatment and devising better methods, or at least pointing out the serious danger of *curetting a puerperal septic uterus.*

When an active puerperal septic condition exists long enough to produce constitutional and local symptoms and signs sufficient to establish a diagnosis, the curette cannot reach the diseased parts, for the germs have extended beyond the endometrium into the uterine wall, blood-vessels and lymphatics, and in rapid septic cases has extended so far that even hysterectomy is not justifiable in but few instances.

The curette not only fails to remove the diseased tissue and germs of infection, but produces serious traumatism, breaks down nature's barriers, dislodges thrombi, increases absorptive surfaces, developing hemorrhage and shock, frequently followed by chills and elevation of temperature.

To these conditions add the use of the gauze tampon, and if the patient recovers it will be in spite of the treatment.

I care not if small portions of placenta or cotyledon be present, *efficient* drainage will eliminate the toxins sufficiently to wait for nature to separate the structures far more efficiently than the curette, when they can easily be removed by forceps without traumatism to parts and with far greater safety to patient.

A gauze tampon does not drain and should never be used in a puerperal septic uterus except to check hemorrhage. It is saturated with blood by the time uterus is packed; the gauze is compressed in uterine cavity, constricted in cervical canal, shutting off capillary current, while the débris, corpuscular elements and germs soon fill its meshes and obstruct drainage, else it would not check hemorrhage. In a few hours the pulse and temperature rise as an index of obstructed drainage; remove the tampon, irrigate uterus, then the pulse and temperature fall as a result of relieved obstruction.

Those that advocate washing out uterus say irrigations should be repeated every few hours. Why? Because the uterus will not drain itself, and in a few hours the toxic elements accumulate in sufficient quantity to produce constitutional symptoms.

Antiseptic irrigation of alcohol, formaldehyde, mercuric chloride, carbolic acid, creolin, iodine in weak or strong solutions, do not take the place of drainage.

Strong solutions fail on the same basis as the curette—they cannot reach all the diseased tissues or germs; they affect only the endometrium, usually coagulating a film upon its surface, and the septic germs play havoc beneath. If they are frequently repeated they interfere with nature's repair of endometrium, set up irritation, favor absorption by producing raw surfaces, and frequently constitutional effects of the drug used.

If weak solutions are used, then they must be often repeated, thus destroying rest of patient; require constant attendance of the physician, and if not properly used, the disturbance of patient, irritation of endometrium, pain and exposure of patient, counterbalance the good effects obtained.

The indiscriminate, reckless use of stimulants in these cases does not remove nor eliminate cause of the disease, and should be relegated to oblivion the same as the indiscriminate, reckless use of opiates and the coal-tar group.

The serum treatment of these cases is on trial, with a hope that some good may result.

So far as the present treatment of these cases is concerned, I consider uterine and alimentary drainage and elimination the most potent factors at our command. Alimentary elimination secured by salines, but not carried to the point of debilitating and weakening the patient, and not too often repeated.

Uterine drainage is most important, and is secured by use of drainage-tubes and strips of gauze or wicking.

The drainage-tubes may be of soft rubber, hard rubber, glass, or aluminum. So far I have had best success with soft rubber tubes, the next best being hard rubber, both made as seen in cuts—black or red.

FIG. 1. SOFT RUBBER.

FIG. 2. HARD RUBBER.

Use the *best quality* (black or red) of soft rubber tubing, as large as cervix will admit, usually one-half ($\frac{1}{2}$) inch in diameter, cut so as to be long enough to reach fundus and protrude 1 to $1\frac{1}{2}$ inches from cervix into vaginal canal; cut large openings in intra-uterine portion, two in vaginal portion, one anterior and one posterior, near end of tube. Now cut off a piece of tubing same, size 1 to $1\frac{1}{2}$ inches long, in center of which cut two openings just barely large enough to admit the other tube and fit snugly to prevent slipping.

Place the short tube on the other tube so as to form a —|— just above the openings in vaginal portion. When in position the cross-bar should rest against cervix, and the ends impinge on lateral vaginal walls sufficient to prevent tube slipping out of uterus, and yet not make sufficient pressure on vaginal walls as to produce irritation or ulceration. The length of cross-bar should depend upon width of vagina.

When tube is in position the openings (large and plenty) in uterine portion give free advent of all discharges; the cervical portion, being *without openings*, conducts discharge to the vaginal portion, where it finds free exit through the two lateral and end openings of tube. The two lateral openings are essential to successful drainage, for frequently the end opening becomes obstructed by burrowing in vaginal folds.

If the tube is curved it should be introduced so as to correspond with the curve of parturient canal, whether uterus be anteverted, retroverted or in normal position.

The soft rubber tube being flexible accommodates itself to the parts without any undue pressure, hence its advantage over non-flexible tubes.

Those not accustomed to use of the tube will find the hard rubber tube more convenient and easier handled.

With all instruments sterilized, water boiled, solutions for irrigation ready, patient is placed across bed, hips near edge on a piece of oil cloth, feet and legs protected, resting on chair to either side, with basin between to catch water. Irrigate vagina, washing out discharge, then introduce a Sims or bivalve speculum, Sims usually preferable, expose cervix and steady uterus with tenaculum, introduce steel dilators, hold cervical canal open until uterus is thoroughly irrigated.

Grasp intra-uterine end of tube (soft rubber) with slightly curved uterine-dressing forceps, anoint end only with carbolized vaseline, cervix steadied with tenaculum, carry tube to fundus of uterus, then steady the tube with tenaculum, forceps or finger, and withdraw the uterine-dressing forceps, leaving tube in position.

If capillary drainage is also desired, use a strip of gauze the length of drainage-tube, one end placed in grasp of forceps and carried to fundus with drainage-tube, introducing both at same time, leaving the other end protruding through cervix in vagina.

The tube should be removed and uterus irrigated once or twice in twenty-four hours in severe cases, being governed by pulse and temperature. If they rise it is an indication for irrigation or that that the drainage is obstructed.

After first irrigation and tube worn for twelve to twenty-four hours dilators will not be needed, as cervix will remain open and not obstruct the return current.

Always irrigate vagina, then uterine cavity with *weak*, antiseptic solution or plain hot water ( unless it be at first or second irrigation, for reasons previously stated ) before placing tube. This irrigation washes out any débris or retained toxic material in uterine cavity or vaginal canal, preventing its absorption, and adds to the efficiency of drainage.

The drainage-tube should be soaked in some *very* strong antiseptic solution while irrigation is performed, then rinse in sterilized water before introducing.

*To introduce the uterine drainage-tube by sense of touch without use of speculum is as great a breach of aseptic work as to introduce uterine sound or catheter by touch without cleansing the parts.*

Always irrigate vagina, expose cervix with speculum and irrigate uterus before introducing tube, so as to do aseptic work with as little pain and discomfort to patient as possible.

Locate fundus of uterus in each case, using bimanual examination and catheter of irrigator, bending the catheter to suit uterine curve; by so doing the irrigating catheter can easily be carried to fundus and the drainage-tube guided in the right direction to facilitate its introduction with little pain to patient and less traumatism to parts.

I emphasize these minor parts in detail and the prevention of pain, because I know of physicians who have failed to get good results and produced so much pain that patients have rebelled and refused to allow them to carry out treatment, which was due to their faulty technique and methods, and not to the principles and results of uterine drainage properly utilized.

As an irrigator always use a metal male catheter of one piece, about No. 17 or 18 French scale with moderate curve, to suit position of uterus. A double curve will frequently facilitate its introduction.

Avoid soft rubber or linen catheters, as they frequently carry infection, being easily roughened, cracked, at times broken, and cannot be properly disinfected. The metal one can be boiled, heated or put in strong antiseptic solutions.

Slip the metal catheter into tubing of fountain syringe, or connect it by a short piece of rubber tubing to an ordinary household syringe, which completes the outfit, all being rendered aseptic before use.

All instruments used with septic case should be left with patient for subsequent use, and sterilized when taken away.

As to drainage in cases of incomplete abortion, there are different conditions to deal with. We limit the term abortion in this paper to interruption of gestation any time prior to the complete formation of placenta. The uterus is small, contracts readily, cavity small, endometrium hypertrophied, placental site less vascular, the decidua serotina more adherent, smaller bulk, more difficult to separate or remove, greater demand for instrumental interference, cervix less obliterated, os smaller, parts less vascular with lessened chances of infection, with a lessened metamorphosis of tissue requiring absorption by lymphatics to complete involution; hence less demand for elimination and drainage than at full term labor.

At least 90 per cent. or more of cases of infection occurring during or after abortion are putrid infection; hence easier controlled and less demand for drainage.

To relieve such, clear and wash out uterine cavity antiseptically, then tampon with gauze. The tampon in these cases is used to check hemorrhage after removal of maternal structures by use of curette or forceps; it also acts as a surgical dressing, protecting denuded area as well as stimulating uterine contractions. If portion of the gauze is saturated with pure camphorated phenol (gum-camphor 2 parts, carbolic acid, pure crystals, 1 part) it acts as an antiseptic, prevents further infection of parts and further elaboration of the putrid toxin.

If an active septic infection occurs then drainage is demanded, even in cases of abortion. Such cases are diagnosed by the *severe* constitutional and local symptoms with rapid inflammatory invasion of the pelvic organs, uterus, tubes, ovaries, lymphatics, blood-vessels and pelvic cellular tissue, part or all.

The dangers and contraindications to use of curette and tampon in septic cases increases in proportion to the advance in pregnancy and the increase in size and vascularity of pelvic organs.

While we advocate an antiseptic gauze uterine tampon after curetting the uterus in cases of abortion, it is to act as a surgical dressing, prevent further infection, check hemorrhage, stimulate uterine contractions, and not for purpose of drainage.

The tampon should be removed in twenty-four to forty-eight hours, and not repeated.

If, after gauze is removed, there is elevation of pulse and temperature, with constitutional and pelvic disturbances, then drainage and elimination are indicated.

The diet and constitutional treatment of these cases do not come within the scope of this paper.

---

### FEVER MIXTURE FOR A CHILD.

The combination given here will act most efficaciously in reducing the temperature of a child in those cases in which no etiologic factor is discoverable:

| | | |
|---|---|---|
| ℞ | Tr. aconiti | gtt. v. |
| | Potassii bromidi | ʒ ss. |
| | Spt. etheris nitrosi | ʒ ij. |
| | Mist. potassii citratis | ℥ ij. |

M. Sig.: Teaspoonful every three hours.

—*Canadian Practitioner.*

## Figures and Tables

**Fig. 1. f1:**
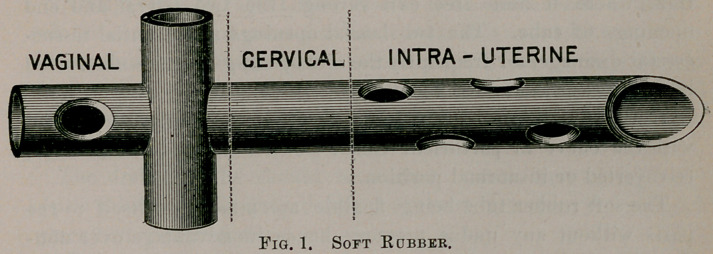


**Fig. 2. f2:**